# Prevalence and Diversity of *Staphylococcus aureus* in Bulk Tank Milk from Community-Based Alpine Dairy Pastures in Tyrol, Austria

**DOI:** 10.3390/ani15142153

**Published:** 2025-07-21

**Authors:** Nasrin Ramezanigardaloud, Igor Loncaric, Patrick Mikuni-Mester, Masoumeh Alinaghi, Monika Ehling-Schulz, Johannes Lorenz Khol, Tom Grunert

**Affiliations:** 1 Centre of Pathobiology, Department of Biological Sciences and Pathobiology, University of Veterinary Medicine, 1210 Vienna, Austria; nasrin.ramezani@vetmeduni.ac.at (N.R.); igor.loncaric@vetmeduni.ac.at (I.L.); masoumeh.alinaghihossein@vetmeduni.ac.at (M.A.); monika.ehling-schulz@vetmeduni.ac.at (M.E.-S.); 2 Centre for Food Science and Veterinary Public Health, Clinical Department for Farm Animals and Food System Science, University of Veterinary Medicine, 1210 Vienna, Austria; patrick-julian.mester@vetmeduni.ac.at; 3 VetmedRegio Tyrol, Clinical Centre for Ruminant and Camelid Medicine, University of Veterinary Medicine, 1210 Vienna, Austria; johannes.khol@vetmeduni.ac.at

**Keywords:** *Staphylococcus aureus*, dairy cows, bulk tank milk, Alpine dairy pastures, subtyping, *spa* typing, DNA-microarray, CC8bov, mastitis

## Abstract

*Staphylococcus aureus* frequently causes intramammary infections in dairy cows (bovine mastitis), which impair animal welfare, milk yield, and food safety. This study determined the prevalence and genetic diversity of *S. aureus* in bulk tank milk (BTM) samples from community-based Alpine dairy pastures in Tyrol, a major milk-producing region in Austria. Throughout the 2023 Alpine season (May–September), 60.3% (94/156) of BTM samples tested positive for *S. aureus* at least once over the course of up to four samplings. A total of 140 isolates collected from the 94 *S. aureus*-positive community-based Alpine dairy pastures revealed 33 distinct *spa* types, with t2953 (*n* = 33), t529 (*n* = 12), t267 (*n* = 11), and t024 (*n* = 10) being the most common. Selected isolates representing the different *spa* types were characterised by DNA microarray-based genotyping, multi-locus sequence typing (MLST), and antimicrobial susceptibility testing. Isolates with *spa* types associated with bovine-adapted CC8 (CC8bov/GTB) were identified as the most common subtype, being detected in BTM samples from 35.3% (55/156) of the pastures. This emphasises the high prevalence of this subtype in dairy herds across European Alpine countries. Other common bovine-associated subtypes were also detected, including CC97, CC151, and CC479. While antimicrobial resistance was rare, enterotoxin-producing genes were detected in all CC8bov-associated *spa* types. Overall, these findings underscore the importance of rigorous hygiene practices in dairy farming, particularly in community-based Alpine dairy pastures, where the risk of transmission is particularly high. It also emphasises the need for continued surveillance and subtyping to improve animal health, ensure food safety, and promote sustainable milk production.

## Simple Summary

Healthy dairy cows are crucial for producing high-quality milk, ensuring food safety, and promoting sustainable milk production. However, udder infections (bovine mastitis), which are frequently caused by the Gram-positive bacterial pathogen *Staphylococcus (S.) aureus*, are among the most common diseases in dairy cows. These infections are costly for farmers, reduce milk production, and raise concerns for cow health and milk safety. Because *S. aureus* spreads from cow to cow, it poses a particular challenge to community-based Alpine dairy pastures. Sharing grazing areas, stables, and milking infrastructure among animals from different farms raises the risk of cross-infection. Here, we collected bulk tank milk samples from 156 community-based Alpine dairy pastures in Tyrol, Austria, throughout the 2023 Alpine season to investigate the prevalence and genetic diversity of *S. aureus* circulating in these farms. We found at least one instance of *S. aureus* in 60% of these pastures. Furthermore, genotyping revealed that a specific subtype of *S. aureus* was widespread, detected in approximately 35% of the pastures, highlighting its significant presence in the European Alpine region. These findings emphasise the need for effective control measures to prevent bovine mastitis caused by *S. aureus*, which is vital for ensuring animal health, sustainable dairy farming, and producing safe food.

## 1. Introduction

*Staphylococcus (S.) aureus* presents a significant threat to human and animal health. In dairy cows, *S. aureus* is a primary cause of intramammary infections (IMIs) globally, negatively impacting animal welfare, food safety, and dairy production. Bovine IMI caused by *S. aureus* is predominantly characterised by subclinical, chronic persistent infections that are difficult to treat. *S. aureus* strains can be grouped into clonal complexes (CCs) based on their phylogenetic relationship, which may differ in their ability to adapt to the host. While some CCs demonstrate strict host specificity, others display remarkable adaptability across species barriers [1]. Among bovine-associated lineages, CC151 and CC97 are the most widely distributed globally [2]. CC151 is an archetypal bovine-specific lineage, whereas CC97 exhibits a broader host range, primarily being isolated from bovine but also from human hosts. In the European Alpine region, the bovine-adapted CC8 variant (CC8bov), also referred to as Genotype B (GTB), has been frequently isolated from dairy cows with IMI [3]. CC8 exemplifies interspecies transmission, as evidenced by a recent human-to-bovine host jump event [4,5]. Both lineages, CC97 and CC8bov, highlight the significance of *S. aureus* as a zoonotic pathogen.

Phylogenetic grouping into CCs has been correlated with key phenotypic traits, including biofilm formation capacity, cellular internalisation capabilities, transmission dynamics, and antimicrobial resistance profiles. To improve the prevention, detection, and treatment of *S. aureus* IMI, subtyping techniques such as genotyping could potentially help farmers and veterinarians implement targeted control and treatment strategies.

In Austria, while the bovine-associated lineages CC151 and CC97 have been detected throughout the country, previous studies suggest that CC8bov is primarily present in the western Alpine region [6,7]. In Vorarlberg, Austria’s westernmost province, an analysis of bulk tank milk (BTM) samples revealed that 10 out of 18 dairy farms were positive for *S. aureus*, with CC8bov being present in half of the positive cases [6]. Studies in Swiss dairy farms also reported a high prevalence of CC8bov, with cow prevalence reaching up to 87% (median 47%) and herd prevalence at 37% [8,9,10]. CC8bov is considered highly contagious, with its transmission largely being attributed to cow movements between herds and the sharing of milking equipment within the same herds [11,12]. This poses a particular challenge for Alpine farming systems, where animals from different farms are commingled on pastures, sharing grazing areas, stables, and milking infrastructure, which increases the risk of transmission. Moreover, CC8bov can enter the dairy production chain, especially raw milk products, posing a potential health risk to consumers due to its secretion of heat-stable enterotoxins [6,13].

In this study, we examined the prevalence and diversity of *S. aureus* in BTM samples from community-based pastures located in the western Austrian Alpine region, in the federal province of Tyrol. Tyrol accounts for the largest proportion of milking pastures (53.7%, 2018) and Alpine dairy cows (62%, 2018) in Austria [14]. Investigating the prevalence and diversity of *S. aureus*, particularly the CC8bov variant, is essential for improving animal welfare and ensuring consumer safety in the context of dairy production.

## 2. Materials and Methods

### 2.1. Sampling

During the summer season in the Alpine region of western Austria, including Tyrol, dairy farming practices incorporate a communal grazing system where dairy cows from several herds are brought together on high-altitude common pastures. These community-based pastures provide shared access to grazing areas, stables, and milking infrastructure. As part of the routine monitoring programme of the Tyrol Milch dairy, all shared pastures that host dairy cows from at least two participating farms and deliver milk to the facility in Wörgl were examined (*n* = 163). The selection of these pastures was not influenced by any prior information, such as clinical history and herd status. Traditionally, the summer grazing period extends from May/June to late August to mid-September, depending on weather conditions. While the initial plan outlined three sampling time points (beginning, middle, and end of the season), unfavourable weather conditions in 2023 delayed the transfer of livestock to the high-altitude communal pastures. This delay necessitated the addition of an extra sampling point between the start and midpoint of the season. In total, we collected 465 bulk tank milk (BTM) samples from 163 pastures across four time points between late May and early September 2023, as follows: first sampling—30–31 May; second sampling—4–5 July; third sampling—9–10 August; and fourth sampling—6–7 September. Most BTM samples were collected from pastures in replicates with varying sampling frequencies, as follows: 26.9% (*n* = 42) were sampled four times from June to September 2023, 48.1% (*n* = 75) were samples three times, 21.2% (*n* = 33) were samples twice, and 3.9% (*n* = 6) were sampled once.

### 2.2. Bacterial Isolation and Identification

Samples were transported to the laboratory in sterile tubes maintained at 4 °C. Because many samples were collected on the respective sampling dates, they were stored at −20 °C pending analysis. Bacterial enrichment was conducted using Matrix-Lysis, a sample preparation method recognised for its simplicity, reliability, and cost-effectiveness in detecting pathogenic bacteria in food matrices [15]. Briefly, 20 mL of milk was combined with lysis buffer (comprising 1 M Tricin pH 7.4 and 1 M MgCl_2_) to a final volume of 40 mL. The mixture was homogenised by vigorous shaking and incubated at room temperature for 15 min. Subsequently, samples were centrifuged at 32,200 × g for 30 min, and the remaining pellet was resuspended in 1000 µL PBS. A 100 µL aliquot was used to culture viable bacteria on Columbia Nalidixic Acid agar (BD Columbia CNA Agar with 5% Sheep Blood, Improved II; C = colistin, *n* = nalidixic acid, A = aztreonam) at 37 °C for 18–24 h. Up to four presumptive, morphologically distinct staphylococci colonies were subcultured onto mannitol salt agar, identified using matrix-assisted laser desorption ionisation–time-of-flight mass spectrometry (MALDI-TOF MS) (Bruker Daltonik, Bremen, Germany), and *S. aureus* was confirmed via the detection of the *nuc* gene [16].

### 2.3. Molecular Subtyping and Microarray

All *S. aureus* isolates were genotyped by *spa* typing [17]. Sample preparation and *spa* sequence typing were performed as previously described [18]. For *spa* typing, the polymorphic X-region of the protein A (*spa*) was amplified and sequenced according to the Ridom Spa Server protocol (https://spa.ridom.de/, last accessed on 25.01.2025). *Spa* types were determined using Ridom SeqSphere + Software v8.4 (Ridom, Münster, Germany) and the Ridom SpaServer website (http://www.spaserver.ridom.de, last accessed on 25.04.2024). One isolate per detected *spa* type (*n* = 33) was chosen randomly and subjected to DNA microarray-based technology (INTER-ARRAY Genotyping Kit *S. aureus*, Bad Langensalza, Germany) to detect antimicrobial resistance and virulence genes [19], as well as being subjected to multi-locus sequence typing (MLST) [20] to identify the respective ST type and clonal complex (CC). The visualisation of the DNA microarray data was performed using a SplitsTree4 phylogenetic network tool [21]. In addition, to gain an overview of the distribution of CCs across community-based Alpine dairy pastures, we inferred all isolates of a specific *spa* type as belonging to the same CC, based on the strong, established correlations between known *spa* types and specific MLST-defined CCs within the *S. aureus* population structure [19,22,23].

### 2.4. Antimicrobial Susceptibility Testing

Antimicrobial susceptibility testing (AST) was conducted using the agar disc diffusion test according to the Clinical and Laboratory Standards Institute (CLSI M100, 2025), testing for the following antimicrobial agents: penicillin (PEN 10 units), cefoxitin (FOX 30 μg), ciprofloxacin (CIP 5 μg), gentamicin (GEN 10 μg), tetracycline (TET 30 μg), erythromycin (ERY 15 μg), clindamycin (CLI 2 μg), chloramphenicol (CHL 30 μg), trimethoprim-sulfamethoxazole (SXT 1.25/23.75 μg), nitrofurantoin (NIT 300 μg), rifampicin (RIF 5 μg), and linezolid (LZD 30 μg) (Beckton Dickinson (BD); Heidelberg, Germany). *S. aureus* ATCC^®^ 25,923 was used as a quality control strain.

### 2.5. Moran’s I Spatial Autocorrelation

Spatial analysis was performed on *S. aureus* prevalence data to assess the spatial autocorrelation across the study area. A data point was classified as positive if *S. aureus* was detected in BTM at least once during the season. The geographic location of each pasture was based on latitude and longitude coordinates. Global and Local Moran’s I statistics were calculated in Python (v3.11) using the GeoPandas v1.0.1, PySAL’s ESDA v2.7.0, libpysal v4.12.1, and Matplotlib libraries v3.8.4. The k-nearest neighbours (k = 4) approach was used to define the spatial weights matrix with row standardisation. The statistical significance was assessed by Monte Carlo permutation with 999 simulations. Global Moran’s I provided a single measure of spatial dependence across the study area, while the use of the Local Moran’s I is important to identify patterns of spatial association. A Moran’s I cluster map highlights areas of high or low spatial autocorrelation based on different categories (e.g., clusters of high values, clusters of low values, etc.) [24]. The Local Moran’s I cluster map categorises observations into four types, as follows: High–High (HH)—areas with high presence surrounded by other high presence (hot spot); Low–Low (LL)—areas with low presence surrounded by other low presence (cold spot); High–Low (HL)—areas with high presence surrounded by low presence (spatial outliers, hot spot in a cold region); Low–High (LH)—areas with low presence surrounded by high presence (spatial outliers, cold spot in a hot region).

## 3. Results

### 3.1. Prevalence of S. aureus in BTM Samples of Pastures

**Figure 1:**
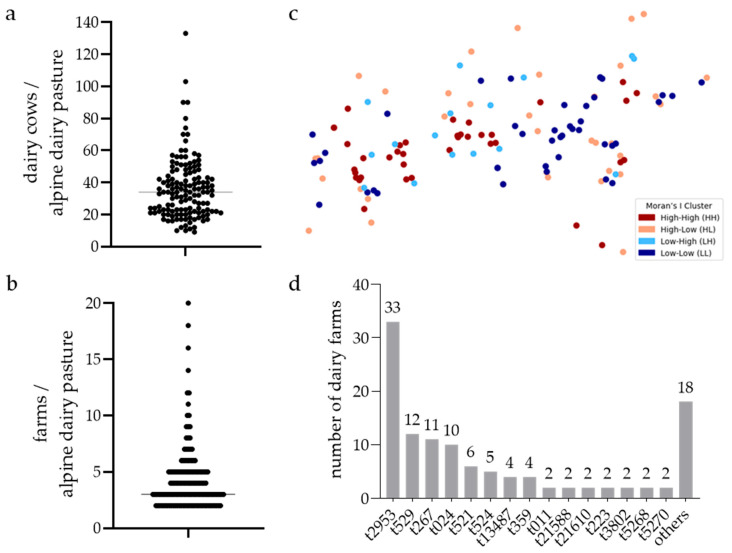
(**a**,**b**) Dot plots illustrating the variation in the number of (**a**) dairy cows and (**b**) farms per community-based Alpine dairy pasture examined. Each dot represents an individual Alpine dairy pasture; the horizontal line indicates the median value. (**c**) Local Moran’s I cluster map showing the spatial autocorrelation of *S. aureus* in community-based Alpine dairy pastures in Tyrol. The colours denote four spatial clusters derived from Local Moran’s I analysis based on similarity or dissimilarity to surrounding points, as follows: High–High clusters (HH, red) indicate hotspots—positive points surrounded by positive points; Low–Low clusters (LL, dark blue) represent coldspots—negative points surrounded by negative points; Low–High (LH, light orange) and High–Low (HL, light blue) show spatial outliers—negative points surrounded by positive points or positive points surrounded by negative points, respectively. (**d**) Distribution of *spa* types across surveyed community-based Alpine dairy pastures. Number of pastures where a particular *spa* type was detected at least once during the season.

To assess the presence of *S. aureus* at the farm level, we analysed 156 community-based Alpine dairy pastures from June to September 2023. The median herd size was 34 dairy cows per pasture, with a range of 9 to 133 cows (Figure 1a), totalling around 5500 dairy cows across all participating pastures. Each community-based Alpine dairy pasture received dairy cows from a median of 3 farms (ranging from 2 to 20, Figure 1b).

Throughout the 2023 season, 60.3% of the pastures (94 out of 156) had at least one BTM sample test positive for *S. aureus*. The first sampling detected 34.9% of Alpine dairy pastures to be positive (30 out of 86 samples), the second sampling detected 31.7% (46 out of 145 samples), the third sampling detected 33.8% (42 out of 124 samples), and the fourth sampling detected 17.3% (19 out of 110 samples). Notably, a lower percentage of *S. aureus*-positive BTM samples was detected on the last sampling date, despite a similar total sample count.

Next, we used Moran’s I analysis to statistically assess if the spatial distribution of pastures with positive *S. aureus* BTM samples was random or showed clustering. The global Moran’s I indicated a weak but statistically significant spatial autocorrelation in the data (I = 0.0912, *p*-value = 0.034). The Moran’s I cluster map identified geographic areas where the spatial clustering of pastures with positive *S. aureus* BTM samples exists (Figure 1c).

### 3.2. Diversity of S. aureus Isolates in BTM Samples of Pastures

We used *spa* typing to subtype all 140 isolates collected from the 94 *S. aureus*-positive community-based Alpine dairy pastures. Thereby, 33 distinct *spa* types were identified, comprising eight previously unreported *spa* types: t21587, t21588, t21610, and t21743-47. The most frequently detected *spa* types included t2953 (*n* = 33), t529 (*n* = 12), t267 (*n* = 11), and t024 (*n* = 10) (Figure 1d). A single *spa* type was identified in most pastures (79%; 78/94). Two distinct *spa* types were present in 19% (18/94), and three *spa* types were found in 2% (2/94) of the pastures, respectively.

### 3.3. DNA Microarray-Based Genotyping, MLST, and Antimicrobial Susceptibility Testing of Selected Isolates

**Figure 2:**
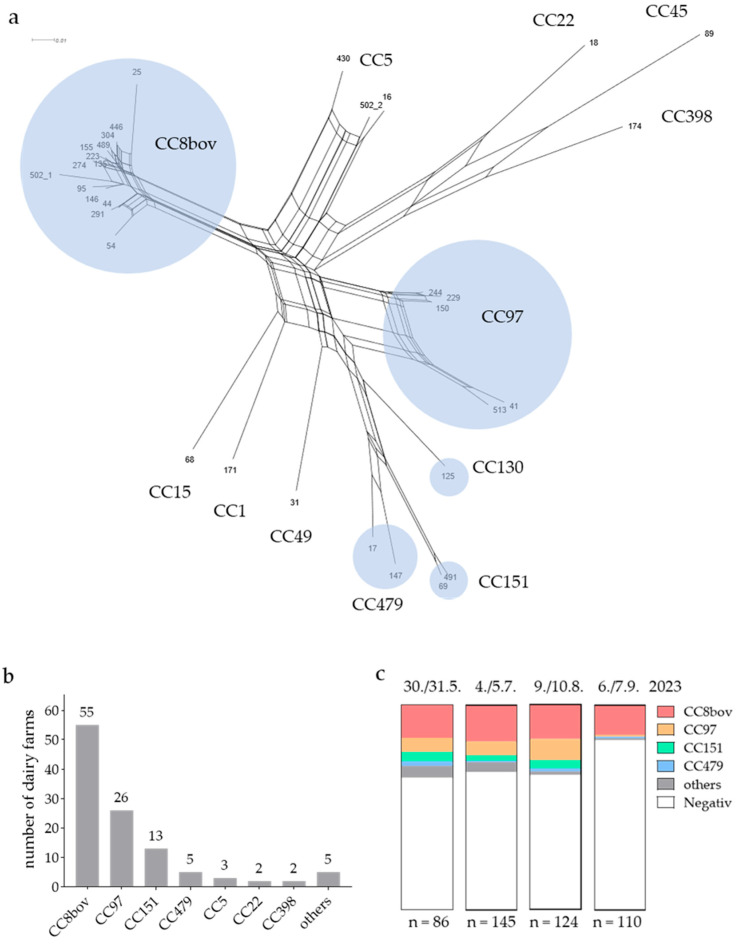
(**a**) SplitsTree network visualisation showing the genetic relationships among the detected *spa* types from *S. aureus* BTM isolates, as determined by DNA microarray hybridisation patterns. Isolates in closer proximity to each other are more closely phylogenetically related, with clustered strains belonging to the same CC. Bovine-associated CCs are highlighted in blue. (**b**,**c**) Distribution of CCs across surveyed community-based Alpine dairy pastures. (**b**) Number pastures where a particular CC was detected at least once during the season. (**c**) The relative proportion of CCs at four sampling time points between the end of May and the beginning of September 2023. N indicates the number of pastures sampled at a particular sampling time point.

One isolate per *spa* type (*n* = 33) was subjected to DNA microarray-based genotyping, multi-locus sequence typing (MLST), and antimicrobial susceptibility testing. SplitsTree analysis used the microarray data of more than 330 genetic markers, revealing several distinct clonal groups, which were categorised according to the similarity of DNA microarray profiles (Figure 2a).

Using MLST, all isolates of a distinct clonal group could be aligned to a specific CC. Notably, 14 different *spa* types were assigned to CC8bov, revealing high *spa* gene diversity for this CC. *Spa* type t2953 represented the most dominant (*n* = 33), followed by t024 (*n* = 10), while the remaining twelve *spa* types assigned to CC8bov were detected sporadically (*n* = 1 or 2). These include seven new *spa*-types (t21587, t21588, t21610, t21743, t21745, t21746, and t21747). During the season, *S. aureus* isolates with *spa* types associated with CC8bov were detected at least once in BTM samples from 35.3% (55/156) of all pastures, making it the most prevalent clonal complex. CC97 and CC151 were detected in 16.7% (26/156) and 8.3% (13/156) of pastures, respectively, followed by CC479, at 3.2% (5/156) (Figure 2b). Notably, while the prevalence of CC97 and CC151 in BTM samples decreased at the last sampling point, the prevalence of CC8bov remained relatively stable across all four samplings (Figure 2c).

Among the genetic markers tested by DNA microarray (*n* = 33 isolates; 1 isolate per *spa* type), we focused on the enterotoxin and leukocidin genes due to their potential relevance to *S. aureus* food intoxication and virulence, respectively (Table 1).

All examined *spa* types of CC8bov (*n* = 13) contained enterotoxin genes in one of the following three variants: either the *sea* gene alone; *sed*, *sej*, and *ser*; or in combination with the *sea* gene. In CC97, one *spa* type (*t359*) was found to carry the *sea* gene. The enterotoxin gene cluster *egc*, comprising the genes *seg*, *sei*, *sem*, *sen*, *seo*, and *seu*, was associated with CC97, CC151, and CC479. A single isolate (*t223*, CC22) carried the human variant of the toxic shock syndrome toxin 1 gene (*tst-1*). None of the isolates exhibited the *sec* gene.

We found the following five leukocidin gene profiles: *lukMF’*-*lukED-lukFS*; *lukED*-*lukFS*; *lukD-lukFS*; *lukED*-*lukS*; and *lukFS*. LukMF’, also referred to as bovine LukM/LukF-P83, explicitly targeting bovine leukocytes, was detected in the combination *lukMF’*-*lukED-lukFS* associated with CC49, CC97, CC151, and CC479. In contrast, *lukFS*-*lukED* (without *lukMF’*) was associated with the lineages CC1, CC5, CC8bov, CC15, and CC97. All CC8bov harboured the leukocidin genes *lukFS*-*lukED*.

All examined BTM isolates (one isolate for each *spa* type, *n* = 33) were susceptible to cefoxitin, and the *mecA* or *mecC* genes were not detected (Table 1). In total, 29 out of 33 isolates were susceptible to all tested antimicrobial agents—penicillin, cefoxitin, ciprofloxacin, amikacin, gentamicin, tetracycline, erythromycin, clindamycin, chloramphenicol, trimethoprim-sulfamethoxazole, nitrofurantoin, rifampicin, and linezolid. Four isolates were resistant to penicillin mediated by the *bla* operon genes (*blaZ*, *blaI*, and *blaR*), associated with CC5, CC15, CC45, and CC398. One isolate (CC398) was resistant to tetracycline mediated by the *tet*(K) gene. One isolate (CC5) was resistant to erythromycin (*erm*(A) gene-positive) with a positive D-zone test (inducible clindamycin resistance). These four isolates, showing phenotypic resistance, were associated with lineages that were non-typical for bovine-associated CCs (CC5, CC15, CC45, and CC398). Interestingly, 12 out of 14 isolates of CC8bov were positive for all *bla* genes but exhibited no phenotypic resistance to penicillin—a discrepancy just recently reported for this CC [25].

## 4. Discussion

The results of our in-depth monitoring of *S. aureus* in Alpine dairy pastures in Tyrol underline the importance of *S. aureus* surveillance, especially in community-based Alpine dairy pastures. Consistent with reports from other European Alpine regions [6,10,26], we found a high prevalence of *S. aureus* (60% in BTM samples) in community-based Alpine dairy pastures in Tyrol. Generally, the reported prevalence of *S. aureus* in BTM samples from dairy herds exhibits significant regional variations, ranging from 18% (Lower Saxony, Germany) [27] to 84% (Minnesota, U.S.) [28]. Apart from the region, the prevalence of the contagious mastitis-causing pathogen *S. aureus* can be influenced by various factors, such as milking procedures, housing systems, herd size, and season [27,29,30]. Moreover, it is important to note that methodological differences, including sampling intensity and (pre-) analytics, can significantly influence the reported prevalence of *S. aureus* in BTM samples. In our study, samples were collected up to four times throughout the season, which likely contributes to a higher detection sensitivity compared to studies employing less-frequent or single-farm samplings, which should be considered when relating our findings with reported prevalence rates from other studies.

The high prevalence of *S. aureus* in community-based Alpine dairy pastures presents a significant challenge in preventing the transmission of *S. aureus* during the Alpine grazing season. The higher presence of *S. aureus* observed in BTM samples in close geographic proximity indicates that factors such as the movement of animals, personnel, or shared equipment between neighbouring farms may influence transmission. As only BTM samples from community-based pastures were included in the study, the local clustering is not surprising but rather highlights the increased risk of transmission when cows from different farms share communal pastures. Rigorous hygiene practices, such as intermediate disinfection and the correct maintenance of milking equipment, are important measures to prevent the spread of *S. aureus* among farms sharing community-based Alpine dairy pastures.

Overall, our study confirms the high prevalence of CC8bov in dairy farming within the European Alpine region. CC8bov was detected in 35.3% of BTM samples during the season, which is comparable to the 36.9% reported in dairy herds in Ticino, Switzerland [10], and 29.3% in Lombardy, northern Italy [31]. The predominant lineages detected were CC8bov, followed by CC97, CC151, and CC479; collectively, these represented nearly 90% of *S. aureus* lineages in BTM samples. These lineages are also among the six most frequently associated with bovine IMI in Europe [3]. CC8bov, CC97, and CC151 were also most common in BTM samples in Lombardy, Italy [31]. CC479 has been detected in cows with IMI in Germany, Belgium, the Netherlands, Portugal, and Italy [3]. Notably, the fourth sampling at the end of the season showed a much lower prevalence of *S. aureus* (17.3%) compared to the first three (average: 33.5%). This decline appears to be associated with a shift in the relative composition of the *S. aureus* lineages in the BTM samples. Specifically, the prevalence of lineages CC97 and CC151 decreased compared to previous samplings, whereas that of CC8bov remained stable. The continuous detection of CC8bov throughout the season demonstrates its critical role in the herd management of Alpine dairy pastures, including efforts for eradication [10]. However, it remains elusive whether there is any causal relationship between the decline in *S. aureus* prevalence in the last sampling and the distribution of the lineages. Several factors may influence this observed correlation. For example, consultations with regional farmers and veterinarians confirmed a common practice, whereby cows exhibiting clinical signs or other health issues with adverse effects on milk production are preferentially selected for dry-off and receive intramammary antibiotic treatment towards the end of the grazing season as the calving season approaches. It is tempting to speculate that management decisions may be biassed by the lineage; however, this hypothesis needs to be investigated further. Isolates from BTM samples may also originate from extramammary sites, such as teat skin, the environment, or personnel operating on the farm [31,32]. Consistent with this, the remaining rarely detected lineages (including CC5, CC22, CC398, and CC45) are associated with colonisation and the infection of humans, as well as pig farming (CC398) [33].

Beyond lineage classification, the isolates also exhibited distinct profiles of virulence factors and antibiotic resistance. Among the leukocidin genes, eight isolates of different CCs harboured the *lukMF*’ operon, which is particularly associated with bovine isolates. LukMF’ can lyse bovine neutrophils and monocytes, and it is a significant virulence factor associated with the severity of *S. aureus* IMI [34]. Leukocidin genes encoding Panton-Valentine leukocidin (PVL, *lukF-PV* and *lukS-PV*) were not detected, which is unsurprising as they are rarely found in bovine isolates and are primarily linked to human *S. aureus* infections [35].

Among the enterotoxin genes, *sea* and *sed* are most frequently associated with human foodborne outbreaks, including those involving dairy products. These most relevant enterotoxins were detected in all 13 *spa* types of CC8bov examined. As we showed previously, CC8bov can enter BTM from cows with subclinical, mild IMI and persist through dairy processing and cheese production, remaining viable in cheese for up to 14 days of ripening [6]. This suggests that CC8bov appears to be particularly well adapted to the cheese production environment. Due to their production of enterotoxins, these *S. aureus* strains pose a potential health risk to consumers. For instance, an *S. aureus* strain with CC8bov characteristics, isolated from soft cheese, was linked to a foodborne outbreak at a Swiss boarding school [36]. In Austria, a CC8bov strain was linked to an outbreak among elementary school children in Lower Austria related to milk products [37]. Thus, reducing *S. aureus* contamination at the source enhances food safety by preventing potential toxin-producing subtypes from entering the food chain, lowering the risk of foodborne illnesses in consumers.

In our study, no methicillin-resistant *S. aureus* (MRSA) isolates were detected in BTM samples. In general, the prevalence of MRSA in BTM samples from dairy herds is considered low, averaging around 3% [38]. Phenotypic resistance to penicillin, tetracycline, erythromycin, and clindamycin was shared by a small number of isolates (*n* = 5/33) that are associated with the lineages CC5, CC15, CC45, and CC398, which are not typically of bovine origin. All other isolates, including the typical bovine-associated lineages, were susceptible to all antimicrobials tested. Interestingly, most analysed CC8bov isolates were positive for all *bla* genes, although they were susceptible to penicillin. The discrepant result was recently described as being exclusively associated with *S. aureus* CC8bov isolates, in which the promoter of the *bla* operon is inactivated by a 31 bp deletion [39]. Overall, our findings support recent observations that antibiotic resistance is becoming less prevalent in *S. aureus* mastitis isolates from Europe and that bovine mastitis-associated lineages are not a significant source of antimicrobial resistance [25,40].

## 5. Conclusions

This study provides an in-depth insight into the *S. aureus* subtypes circulating in community-based Alpine dairy pastures in Tyrol, a region highly relevant for bovine milk production in Austria. Our findings indicate that *S. aureus*, particularly the CC8bov lineage, is highly prevalent in dairy herds throughout this region, consistent with findings in other European Alpine countries. Additionally, we identified the lineages CC97, CC151, and CC479, all of which are frequently associated with bovine IMI in Europe. Understanding the variability among these lineages could help inform targeted interventions and improve strategies for controlling infections caused by this zoonotic pathogen. Overall, our findings underscore the importance of continuous surveillance and subtyping, revealing the risks posed by antibiotic-resistant and toxin-producing bacteria, thereby enhancing animal health, food safety, and sustainable milk production.

## Figures and Tables

**Table 1:** Summarised molecular characterisation and antimicrobial resistance profiles of one *S. aureus* isolate per detected *spa* type (*n* = 33).

Strain-ID ^(1)^	*spa*	CC	ST	*agr* Type	Antimicrobial Resistance Profile		Toxins/Superantigens	Leukocidins ^(4)^
					Phenotype	Genes Detected		
502_1	t2953	CC8	ST8	agr *I*	NR	*blaZ*	*sed*, *sej*, *ser*	*lukF/S*, *lukD/E*
69	t529	CC151	ST504	agr *II*	NR		*egc* cluster ^(3)^	*lukMF’*, *lukF/S*, *lukD/E*
229	t267	CC97	ST352	agr *I*	NR			*lukMF’*, *lukF/S*, *lukD/E*
304	t024	CC8	ST8	agr *I*	NR	*blaZ*	*sea*, *sed*, *sej*, *ser*	*lukF/S*, *lukD/E*
244	t521	CC97	ST97	agr *I*	NR			*lukMF’*, *lukF/S*, *lukD/E*
41	t524	CC97	ST71	agr *I*	NR			*lukF/S*, *lukD/E*
17	t13487	CC479	ST1380	agr *II*	NR		*egc* cluster ^(3)^	*lukMF’*, *lukF/S*, *lukD/E*
54	t359	CC97	ST97	agr *I*	NR		*sea*	*lukF/S*, *lukD/E*
174	t011	CC398	ST2199	agr *I*	PEN, TET	*blaZ*, *tet*(K), *tet*(M)		*lukF/S*
146	t21588	CC8	ST8	agr *I*	NR	*blaZ*	*sed*, *sej*, *ser*	*lukF/S*, *lukD/E*
44	t21610	CC8	ST6180	agr *I*	NR		*sea*	*lukF/S*, *lukD/E*
18	t223	CC22	ST5974	agr *I*	NR		*tst-1* (human), *egc* cluster ^(3)^	*lukF/S*
291	t3802	CC8	ST7509	agr *I*	NR		*sea*	*lukF/S*, *lukD/E*
274	t5268	CC8	ST8	agr *I*	NR	*blaZ*	*sea*, *sed*, *sej*, *ser*	*lukF/S*, *lukD/E*
155	t5270	CC8	ST8	agr *I*	NR	*blaZ*	*sea*, *sed*, *sej*, *ser*	*lukF/S*, *lukD/E*
16	t002	CC5	ST5	agr *II*	ERY, CLI ^(2)^	*erm*(A)	*egc* cluster ^(3)^	*lukF/S*, *lukD/E*
89	t065	CC45	ST45	agr *I*	PEN	*blaZ*	*egc* cluster ^(3)^	*lukF/S*
68	t084	CC15	ST15	agr *II*	PEN	*blaZ*		*lukF/S*, *lukD/E*
171	t127	CC1	ST1	agr *III*	NR		*seh*	*lukF/S*, *lukD/E*
502_2	t1340	CC5	ST5	agr *II*	NR			*lukF/S*, *lukD/E*
430	t179	CC5	ST5	agr *II*	PEN	*blaZ*	*sed*, *sej*, *ser*, *egc* cluster ^(3)^	*lukF/S*, *lukD/E*
491	t18776	CC151	ST504	agr *II*	NR		*egc* cluster ^(3)^	*lukMF’*, *lukF/S*, *lukD/E*
489	t19341	CC8	ST8384	agr *I*	NR	*blaZ*	*sea*, *sed*, *sej*, *ser*	*lukF/S*, *lukD/E*
31	t208	CC49	ST49	agr *II*	NR			*lukMF’*, *lukF/S*, *lukD/E*
223	t21587	CC8	ST6180	agr *I*	NR	*blaZ*	*sea*, *sed*, *sej*, *ser*	*lukF/S*, *lukD/E*
135	t21743	CC8	ST8	agr *I*	NR	*blaZ*	*sea*, *sed*, *sej*, *ser*	*lukF/S*, *lukD/E*
147	t21744	CC479	ST1380	agr *II*	NR		*egc* cluster ^(3)^	*lukMF’*, *lukF/S*, *lukD/E*
25	t21745	CC8	ST9273	agr *I*	NR	*blaZ*	*sea*, *sed*, *sej*, *ser*	*lukS*, *lukD/E*
446	t21746	CC8	ST8384	agr *I*	NR	*blaZ*	*sea*, *sed*, *sej*, *ser*	*lukF/S*, *lukD/E*
95	t21747	CC8	ST8	agr *I*	NR	*blaZ*	*sed*, *sej*, *ser*	*lukF/S*, *lukD/E*
150	t527	CC97	ST352	agr *I*	NR			*lukMF’*, *lukF/S*, *lukD/E*
513	t528	CC97	ST71	agr *I*	NR		*egc* cluster ^(3)^	*lukF/S*, *lukD/E*
125	t843	CC130	ST2490	agr *III*	NR			*lukF/S*, *lukD*

^(1)^ Selected for MLST and DNA microarray-based technology and antimicrobial susceptibility testing. ^(2)^ Erythromycin-induced resistance to clindamycin. ^(3)^ *egc* cluster comprising *seg*, *sei*, *sem*, *sen*, *seo*, *seu*. ^(4)^ *lukMF’*, also referred to as *lukF-P83*/*lukM* (bovine).

## Data Availability

Data generated and/or analysed during the current study are available from the corresponding author upon reasonable request.

## Abbreviations

The following abbreviations are used in this manuscript:

BTM	bulk tank milk
CC	clonal complex
CC8bov	bovine-adapted CC8 variant
GTB	genotype B
IMI	intramammary infection
MALDI-TOF-MS	matrix-assisted laser desorption ionisation–time-of-flight mass spectrometry
MLST	multi-locus sequence typing

## References

[1] Howden B.P., Giulieri S.G., Wong Fok Lung T., Baines S.L., Sharkey L.K., Lee J.Y.H., Hachani A., Monk I.R., Stinear T.P. (2023). *Staphylococcus aureus* host interactions and adaptation. Nat. Rev. Microbiol..

[2] Campos B., Pickering A.C., Rocha L.S., Aguilar A.P., Fabres-Klein M.H., de Oliveira Mendes T.A., Fitzgerald J.R., de Oliveira Barros Ribon A. (2022). Diversity and pathogenesis of *Staphylococcus aureus* from bovine mastitis: Current understanding and future perspectives. BMC Vet. Res..

[3] Boss R., Cosandey A., Luini M., Artursson K., Bardiau M., Breitenwieser F., Hehenberger E., Lam T., Mansfeld M., Michel A. (2016). Bovine *Staphylococcus aureus*: Subtyping, evolution, and zoonotic transfer. J. Dairy Sci..

[4] Resch G., François P., Morisset D., Stojanov M., Bonetti E.J., Schrenzel J., Sakwinska O., Moreillon P. (2013). Human-to-bovine jump of *Staphylococcus aureus* CC8 is associated with the loss of a β-hemolysin converting prophage and the acquisition of a new staphylococcal cassette chromosome. PLoS ONE.

[5] Sakwinska O., Giddey M., Moreillon M., Morisset D., Waldvogel A., Moreillon P. (2011). *Staphylococcus aureus* host range and human-bovine host shift. Appl. Environ. Microbiol..

[6] Kummel J., Stessl B., Gonano M., Walcher G., Bereuter O., Fricker M., Grunert T., Wagner M., Ehling-Schulz M. (2016). *Staphylococcus aureus* Entrance into the Dairy Chain: Tracking, S. aureus from Dairy Cow to Cheese. Front. Microbiol..

[7] Schabauer A., Pinior B., Gruber C.M., Firth C.L., Käsbohrer A., Wagner M., Rychli K., Obritzhauser W. (2018). The relationship between clinical signs and microbiological species, *spa* type, and antimicrobial resistance in bovine mastitis cases in Austria. Vet. Microbiol..

[8] Fournier C., Kuhnert P., Frey J., Miserez R., Kirchhofer M., Kaufmann T., Steiner A., Graber H.U. (2008). Bovine *Staphylococcus aureus*: Association of virulence genes, genotypes and clinical outcome. Res. Vet. Sci..

[9] Graber H.U., Naskova J., Studer E., Kaufmann T., Kirchhofer M., Brechbuhl M., Schaeren W., Steiner A., Fournier C. (2009). Mastitis-related subtypes of bovine *Staphylococcus aureus* are characterized by different clinical properties. J. Dairy Sci..

[10] Sesso L., Vanzetti T., Weber J., Vaccani M., Riva Scettrini P., Sartori C., Ivanovic I., Romanò A., Bodmer M., Bacciarini L.N. (2024). District-wide herd sanitation and eradication of intramammary *Staphylococcus aureus* genotype B infection in dairy herds in Ticino, Switzerland. J. Dairy Sci..

[11] van den Borne B.H.P., van Schaik G., Lam T.J.G.M., Nielen M. (2010). Therapeutic effects of antimicrobial treatment during lactation of recently acquired bovine subclinical mastitis: Two linked randomized field trials. J. Dairy Sci..

[12] Voelk V., Graber H.U., van den Borne B.H., Sartori C., Steiner A., Bodmer M., Haerdi-Landerer M.C. (2014). A longitudinal study investigating the prevalence of *Staphylococcus aureus* genotype B in seasonally communal dairy herds. J. Dairy Sci..

[13] Hummerjohann J., Naskova J., Baumgartner A., Graber H.U. (2014). Enterotoxin-producing *Staphylococcus aureus* genotype B as a major contaminant in Swiss raw milk cheese. J. Dairy Sci..

[14] Austrian Federal Ministry of Agriculture, Forestry, Environment and Water Management INVEKOS Referenzflächen Österreich 2018. https://geoportal.inspire.gv.at/metadatensuche/FhHKXRQ9/api/records/6d1524bc-f610-486a-be54-ba5c956af068.

[15] Mester P., Schoder D., Wagner M., Rossmanith P. (2014). Rapid Sample Preparation for Molecular Biological Food Analysis Based on Magnesium Chloride. Food Anal. Methods.

[16] Brakstad O.G., Aasbakk K., Maeland J.A. (1992). Detection of *Staphylococcus aureus* by polymerase chain reaction amplification of the *nuc* gene. J. Clin. Microbiol..

[17] Harmsen D., Claus H., Witte W., Rothganger J., Claus H., Turnwald D., Vogel U. (2003). Typing of methicillin-resistant *Staphylococcus aureus* in a university hospital setting by using novel software for *spa* repeat determination and database management. J. Clin. Microbiol..

[18] Loncaric I., Künzel F., Licka T., Simhofer H., Spergser J., Rosengarten R. (2014). Identification and characterization of methicillin-resistant *Staphylococcus aureus* (MRSA) from Austrian companion animals and horses. Vet. Microbiol..

[19] Monecke S., Slickers P., Ehricht R. (2008). Assignment of *Staphylococcus aureus* isolates to clonal complexes based on microarray analysis and pattern recognition. FEMS Immunol. Med. Microbiol..

[20] Enright M.C., Day N.P., Davies C.E., Peacock S.J., Spratt B.G. (2000). Multilocus sequence typing for characterization of methicillin-resistant and methicillin-susceptible clones of *Staphylococcus aureus*. J. Clin. Microbiol..

[21] Huson D.H., Bryant D. (2006). Application of phylogenetic networks in evolutionary studies. Mol. Biol. Evol..

[22] Faria N.A., Carrico J.A., Oliveira D.C., Ramirez M., de Lencastre H. (2008). Analysis of typing methods for epidemiological surveillance of both methicillin-resistant and methicillin-susceptible *Staphylococcus aureus* strains. J. Clin. Microbiol..

[23] Sangvik M., Olsen R.S., Olsen K., Simonsen G.S., Furberg A.-S., Sollid J.U.E. (2011). Age- and gender-associated *Staphylococcus aureus* spa types found among nasal carriers in a general population: The Tromso Staph and Skin Study. J. Clin. Microbiol..

[24] Anselin L., Fischer M., Scholten H.J., Unwin D. (1996). Spatial analytical perspectives on GIS. The Moran Scatterplot as an ESDA Tool to Assess Local Instability in Spatial Association.

[25] Nemati G., Romanó A., Wahl F., Berger T., Rojo L.V., Graber H.U. (2023). Bovine *Staphylococcus aureus*: A European study of contagiousness and antimicrobial resistance. Front. Vet. Sci..

[26] Cortimiglia C., Luini M., Bianchini V., Marzagalli L., Vezzoli F., Avisani D., Bertoletti M., Ianzano A., Franco A., Battisti A. (2016). Prevalence of *Staphylococcus aureus* and of methicillin-resistant S. aureus clonal complexes in bulk tank milk from dairy cattle herds in Lombardy Region (Northern Italy). Epidemiol. Infect..

[27] Kortstegge J., Krömker V. (2024). Prevalence of Contagious Mastitis Pathogens in Bulk Tank Milk from Dairy Farms in Lower Saxony, Germany. Hygiene.

[28] Haran K.P., Godden S.M., Boxrud D., Jawahir S., Bender J.B., Sreevatsan S. (2012). Prevalence and characterization of *Staphylococcus aureus*, including methicillin-resistant *Staphylococcus aureus*, isolated from bulk tank milk from Minnesota dairy farms. J. Clin. Microbiol..

[29] Olde Riekerink R.G., Barkema H.W., Scholl D.T., Poole D.E., Kelton D.F. (2010). Management practices associated with the bulk-milk prevalence of *Staphylococcus aureus* in Canadian dairy farms. Prev. Vet. Med..

[30] Ruegg P.L. (2017). A 100-Year Review: Mastitis detection, management, and prevention. J. Dairy Sci..

[31] Gazzola A., Maisano A.M., Bianchini V., Vezzoli F., Romanò A., Graber H.U., Cremonesi P., Zanardi G., Cappa V., Luini M. (2020). Short communication: Characterization of *Staphylococcus aureus* from bulk tank milk of dairy cattle in Lombardy (northern Italy). J. Dairy Sci..

[32] Haveri M., Hovinen M., Roslof A., Pyorala S. (2008). Molecular types and genetic profiles of *Staphylococcus aureus* strains isolated from bovine intramammary infections and extramammary sites. J. Clin. Microbiol..

[33] Aanensen D.M., Feil E.J., Holden M.T., Dordel J., Yeats C.A., Fedosejev A., Goater R., Castillo-Ramírez S., Corander J., Colijn C. (2016). Whole-Genome Sequencing for Routine Pathogen Surveillance in Public Health: A Population Snapshot of Invasive *Staphylococcus aureus* in Europe. mBio.

[34] Vrieling M., Boerhout E.M., van Wigcheren G.F., Koymans K.J., Mols-Vorstermans T.G., de Haas C.J., Aerts P.C., Daemen I.J., van Kessel K.P., Koets A.P. (2016). LukMF’ is the major secreted leukocidin of bovine *Staphylococcus aureus* and is produced in vivo during bovine mastitis. Sci. Rep..

[35] Antók F.I., Mayrhofer R., Marbach H., Masengesho J.C., Keinprecht H., Nyirimbuga V., Fischer O., Lepuschitz S., Ruppitsch W., Ehling-Schulz M. (2019). Characterization of Antibiotic and Biocide Resistance Genes and Virulence Factors of Staphylococcus Species Associated with Bovine Mastitis in Rwanda. Antibiotics.

[36] Johler S., Weder D., Bridy C., Huguenin M.C., Robert L., Hummerjohann J., Stephan R. (2015). Outbreak of staphylococcal food poisoning among children and staff at a Swiss boarding school due to soft cheese made from raw milk. J. Dairy Sci..

[37] Schmid D., Fretz R., Winter P., Mann M., Hoger G., Stoger A., Ruppitsch W., Ladstatter J., Mayer N., de Martin A. (2009). Outbreak of staphylococcal food intoxication after consumption of pasteurized milk products, June 2007, Austria. Wien. Klin. Wochenschr..

[38] Schnitt A., Tenhagen B.-A. (2020). Risk Factors for the Occurrence of Methicillin-Resistant *Staphylococcus aureus* in Dairy Herds: An Update. Foodborne Pathog. Dis..

[39] Ivanovic I., Boss R., Romanò A., Guédon E., Le-Loir Y., Luini M., Graber H.U. (2023). Penicillin resistance in bovine *Staphylococcus aureus*: Genomic evaluation of the discrepancy between phenotypic and molecular test methods. J. Dairy Sci..

[40] Karell J., Petzl W., Gangl A., Huber-Schlenstedt R., Sorge U.S. (2024). Changes in antimicrobial resistance of *Staphylococcus aureus* in bovine quarter milk samples from southern Germany between 2012 and 2022. J. Dairy Sci..

